# High Pressure Spectroscopic Investigation on Proton Transfer in Squaric Acid and 4,4′-Bipyridine Co-crystal

**DOI:** 10.1038/s41598-017-04980-3

**Published:** 2017-07-05

**Authors:** Zhiwei Ma, Juntao Li, Chunyu Liu, Chenglin Sun, Mi Zhou

**Affiliations:** Key Laboratory of Physics and Technology for Advanced Batteries (Ministry of Education), College of Physics Jilin University, Changchun, 130012 P.R. China

## Abstract

In attempt to the obtain detailed geometric information of proton transfer compound (subsequently denote as SQBP) formed between squaric acid (SQ)and 4,4′-bipyridine(BP), and to investigate the mechanisms of pressure-induced double proton transfer and related structural phase transition, we carried out *in-situ* high pressure Raman spectroscopy of SQBP up to 20 GPa. A solid-solid phase transition together with double proton transfer phenomenon was confirmed by Raman spectroscopy at about 1.5 GPa, and the activation of C = O stretching mode in Raman spectra indicates a square-ring structure of SQ with four symmetric C = O bond formation. These results are further supported by first-principals calculations and *in-situ* high pressure infrared absorption spectroscopy. Additionally, Raman intensity analysis suggests that a higher-order phase transition with planar BP molecular structure occurred in the pressure range of 3~6 GPa. As a result, the π electron delocalization effect in BP dominated the intensity enhancement of C = O stretching mode in SQ. To the best of our knowledge, this is the first time observation of the intensity enhancement of proton donor’s normal modes induced by proton acceptor’s π electron delocalization.

## Introduction

Proton transfer(PT) presents as a ubiquitous component among the most basic processes, which plays an important role in many chemical and biochemical reactions^1, 2^. Its empirical study began with the origin of chemistry. Recently, the investigations on PT mainly focused on the crystal engineering^3, 4^, catalytic reactions^5, 6^, and other relevant fields such as organic ferroelectrics^7, 8^, energetic materials^9–11^, non-linear optical materials^12, 13^, hydrogen storage^14–17^ and pharmaceutical industry^18, 19^. PT is also known as a crucial step in many biochemical processes^20–24^. PT is one of the simplest chemical reactions, ranging from complete transfer from an acidic to a basic moiety to varying degrees of sharing through hydrogen bonds. For hydrogen-bonding systems, the level of interaction between donor and acceptor can vary significantly, from relatively weak to strong with quasi-covalent character. There is also the possibility of disordered hydrogen bonds^25–28^. The PT reaction within a hydrogen-bonding systems, which was found to be barrier less and rapid, has been heavily reported^29–31^.Due to a variety of the applications related to PT, it is of great significance to unveil the detailed mechanism of PT in hydrogen-bonding systems.

As one of the basic independent thermodynamic parameters, the pressure is important for characterizing the state in condensed matter physics^32–34^. It can be effective in changing crystal structure, inter-atomic distance and atomic orbitals overlap. Thus, pressure can be used as a driving force to control the transfer direction and pathway of PT in hydrogen-bonding systems, which provides a new strategy to investigate the intra- and inter-molecular interactions. On the other hand, Raman spectroscopy is widely utilized in both physical and chemistry to provide a fingerprint by which molecules can be identified^35–37^. From an experimental point of view on high pressure research, to elucidate the mechanism of phase transition and the changes of crystal or molecular geometry, corresponding Raman spectroscopy is commonly adopted for structural characterizations^38–40^. Then, *in-situ* high pressure Raman spectroscopy can help us get access to molecular symmetric and geometric information about PT in hydrogen-bonding systems.

PT through hydrogen bonds are often found in co-crystal systems^25, 41, 42^. Co-crystals are generally accepted to be neutral complexes composed of two components bonded by non-covalent inter-molecular interactions. Design and synthesis of co-crystals have been a popular research topic because of fundamental interests in crystal engineering which shows potential applications in many areas of functional solids^43–45^. 1:1 stoichiometric proton transfer complex(SQBP) given by squaric acid (SQ) and 4,4′-bipyridine (BP) is a typical proton-transfer co-crystal. At ambient condition, SQBP comprises a protonated squaric acid cation(SQ^+^) and a 4,4′-bipyridine anion(BP^−^), with possible changes of external parameters such as pressure or temperature, double proton-transfer complex (SQ^2+^BP^2−^)could be obtained^46, 47^. These studies raised the following questions. Firstly, what are the detailed structural changes for both proton donor and acceptor in SQBP from that of their reactants. Secondly, the detailed structural characteristics of SQBP at high pressure should be known.

Therefore, We conducted a comparative study with Raman spectroscopic investigations on SQBP. The detailed molecular geometric changes in proton donor and acceptor are discussed. Using *in-situ* high pressure Raman and IR spectroscopic techniques and first-principal calculations, the structural evolution of pressure-induced double PT phase transition in SQBP are further analyzed. And a possible higher-order phase transition is proposed in the pressure range of 3~6 GPa.

## Experimental and Methods

Samples of squaric acid and 4,4′-bipydine were obtained from Acros Organics and were well-preserved before utilization. Under sufficient stirring, 0.044g (0.397 *mmol*) of colorless squaric acid crystals and 0.062g (0.386 *mmol*) of clear 4,4′-bipydine crystals were dissolved completely in 50 ml hydrothermal solution^47^. Yellow colored crystal was formed while cooling slowly at room temperature for 24 hours.

### Spectroscopic investigations

The high-pressure cell used in this experiment was based on symmetric diamond anvil cell (DAC) having two diamonds with 500 μm culet size. The sample with a small ruby chip (10 μm) was loaded in a 200 μm hole drilled in a 250 μm thick T301 gasket pre-indented to 80 μm thickness. The pressure calibration was done by using ruby fluorescence^48^. Raman measurements were conducted with the Acton Spectra Pro 2500i spectrometer equipped with a liquid nitrogen cooled CCD camera (Pylon: 100B). The 532 nm line of diode laser was employed to excite the sample. The spectral resolution was approximately 1 cm^−1^. The laser power was 3.6 mW and the typical accumulation time for each spectra was 30 s. Frequency calibration of the Raman spectrum was implemented and by using the characteristic 520 cm^−1^ of silicon reference. Liquid argon was used to support quasi-hydrostatic conditions in the Raman measurement. The Raman peaks are fitted with Lorentz line shape function.

*In-situ* high pressure IR measurements were conducted using a Nicolet iN10 FT-IR micro-spectrometer. The micrographs of the samples were obtained using a camera (Canon Eos 5D mark II) equipped on a microscope (Ecilipse TI-U, Nikon). UV-visible absorption spectra were collected by a home-made UV-Visible spectrophotometer from ocean optics.

### First-principals DFT calculations

*ab initio* plan-wave pseudopotential density functional method implemented in the CASTEP code^49^ has been used. The initial structure model was constructed on the basis of reported experimental data^46^.The optimization is not finished until the forces on the atoms are less than 0.01eV/ Å and all the stress components are less than 0.02 GPa. The tolerance in the self-consistent field(SCF) calculation is 5 × 10^−7^eV/atom. The norm-conserving pseudopotential is used to describe interactions between nuclear core and valent electrons. Exchange and correlation effects are described in the scheme of Perdew-Burke-Eruzerhof(PBE) generalized gradient approximation(GGA). Convergence tests give a 2π ×  0.03 Å^−1^ grid spacing and 900eV energy cutoff. With such a choice the error bars of total energies are about 0.2 meV per atom. As the detailed crystal structures of the high pressure phases are not known, the theoretical calculation is not extended to the pressure higher than 2.5 GPa.

## Results and Discussion

### Raman spectra of SQ, BP and their proton-transfer co-crystal (SQBP)

**Figure 1:**
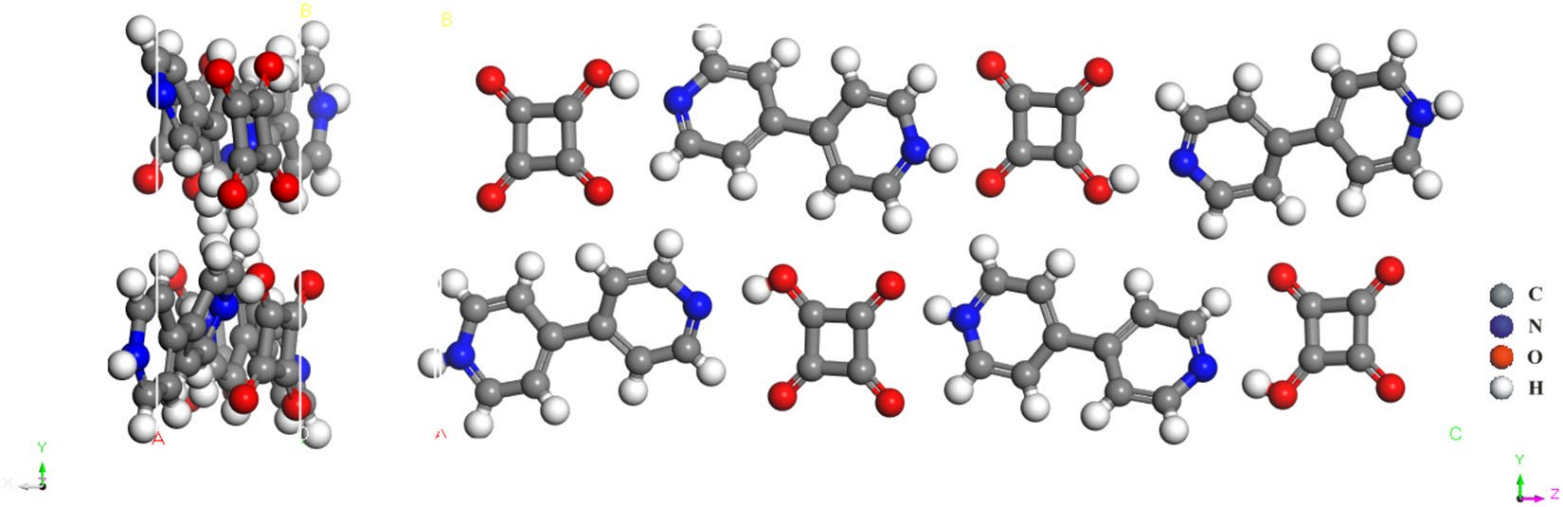
Crystal structure of SQBP at ambient condition.

**Figure 2:**
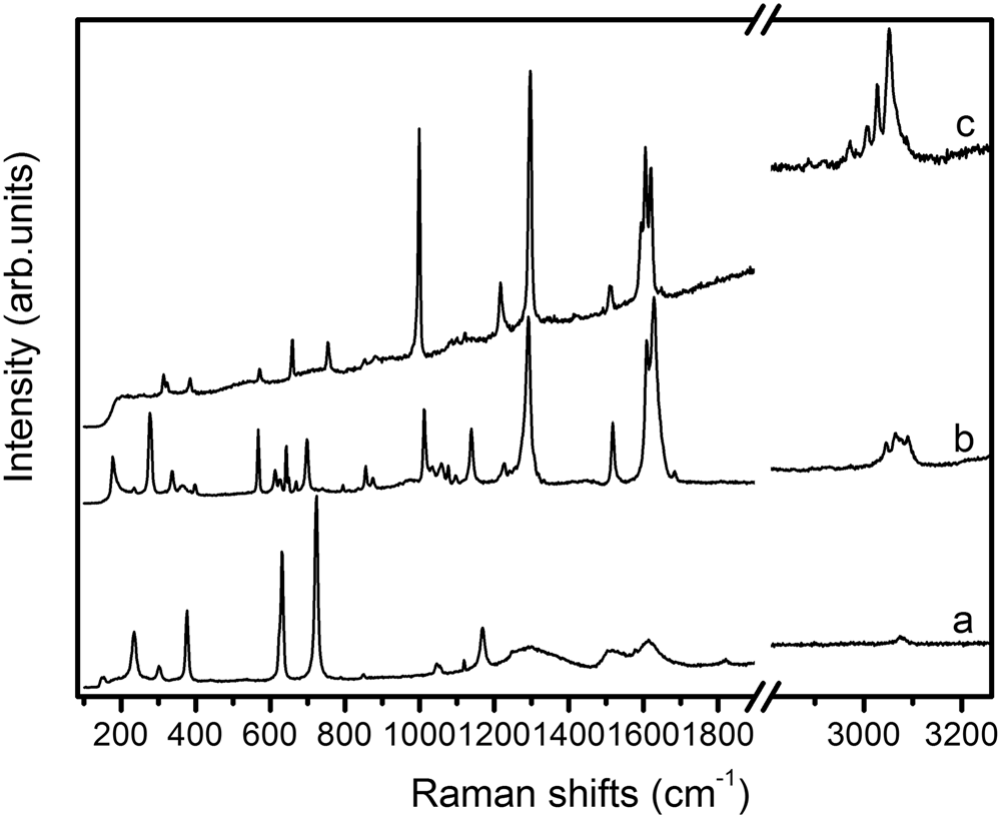
Raman spectra of (**a**), Squaric acid, (**b**), SQBP and (**c**), 4, 4′-bipyridine.

A co-crystal is defined as a crystal that is build up out of two or more different molecular and or ionic compounds. The co-crystal contains the chemical unit of each reactants. At ambient condition, the solid state structure of SQBP has a monoclinic crystal *P*2_1_/*n* with unit cell parameters, *a* = 3.79750(10) Å, *b* = 11.1996(3) Å, *c* = 27.4424(7) Å,ß* = *92.236(2)°, *Z* = 4, *V* = 1166.25(5) Å^3 47^, both SQ and BP in their monoprotonated forms, the crystal structure of SQBP is shown in Fig. 1. Vibrational spectroscopy as a primary source of information on chemical bonding can provide insight into three-dimensional structure of molecules. The Raman spectra of SQ, BP and their proton-transfer co-crystal (SQBP) are shown in Fig. 2. The corresponding peak assignments are listed in Table 1 ^50–53^. It is obvious that the most prominent Raman bands of SQBP are attributed to BP, with some weak bands originating from SQ. Due to the cooperative and competitive effects of hydrogen bonds, the Raman bands of SQBP below 900 cm^−1^ tend to shift to lower wavenumbers in comparison with the reactants, and the corresponding normal modes are attributed to the twisting, bending and ring-ring stretching vibrations of the whole molecular skeleton. This may be due to the attraction of inter-molecular hydrogen bonds, which therefore can expand the molecular skeleton of both BP and SQ. The 328 cm^−1^ band is attributed to the ring-ring stretching vibration of BP, the blue shift of this band indicates that the C-C bond length between pyridine rings is prolonged due to the formation of inter-molecular hydrogen bonds. The aromatic ring-ring stretching mode is used as an indicator of planar molecular geometry of biphenyl and p-terphenyl^54, 55^. The ring breathing mode of SQ shifts from 724 to 698 cm^−1^ in SQBP suggests that the four-membered carbocyclic ring of SQ is expanded in SQBP, as a result the C-C stretching band in SQ moves from 1170 cm^−1^ to 1138 cm^−1^. Compared to the Raman spectra of SQ and BP, at higher wavenumbers, except 1170 and 1295 cm^−1^ bands, all the other Raman lines shift toward the higher wavenumber side. The band at 998 cm^−1^ attributed to the ring breathing vibration of BP shifts to 1012 cm^−1^, which suggests that the pyridine ring shrank slightly in SQBP. As a result, other bands corresponding to the vibrations of pyridine ring also shift toward the higher wavenumber side. Consequently, the expansion of BP’s molecular skeleton is highly likely originated from the change tendencies of Raman bands below 900 cm^−1^. It appears that the expansion of a molecular skeleton and the shrinking of pyridine ring in BP are contradictory, this can be understood from changes in the frequency of inter-ring C-C stretch mode, this band shifts from 1295 to 1290 cm^−1^ suggests that the C-C bond between pyridine rings is lengthened in SQBP. The net result of the shrinking of the pyridine rings and lengthening of the C-C bond is the expansion of BP’s molecular skeleton. There are three distinct bands observed at1595, 1604 and 1619 cm^−1^in the Raman spectrum of BP. We tend to assign the triple bands to a vibrational anharmonic coupling (Fermi resonance) between fundamental C = C stretching mode and a combination band of 658 and 998 cm^−1^. Fermi resonance, a special case of mechanical coupling, is very sensitive to the detailed structure and local environment of the molecule^56, 57^. Due to the intermolecular hydrogen bonds and proton transfer, the molecular structure of BP in co-crystal will generally deviate from its crystal structure. As a result, only two bands are observed at 1609 and 1628 cm^−1^ in the Raman spectrum of SQBP. Moreover, a weak band owing to C = O stretching mode can be found at 1821 cm^−1^ in the Raman spectrum of SQ, while this mode could not be identified in the Raman spectrum of SQBP. There are two possible ways to interpret this spectral phenomenon. Firstly, it is noted that the contribution of SQ to the Raman spectrum of SQBP is small compared with that of BP (Fig. 2), while the weak bands corresponding SQ could not be detected in their co-crystal. Secondly, the C = O stretching mode might become Raman silent due to the molecular symmetry change of SQ in the proton-transfer co-crystal.

**Table 1:** Detailed peaks position and assignments of the SQ, SQBP and BP.

Assignments	Frequency(cm^−1^)		
	squaric acid	1:1 adduct(SQBP)	4,4′-Bipyridine
Ring-ring stretching(BP)		277	328
In-plane CO bending(SQ)	377	366	
Ring twisting(BP)		567	571
Out-of-plane C = O bending(SQ)	624	612	
Ring bending(SQ)	631	625	
Ring bending(BP)		641/649	658
Ring breathing(SQ)	724	698	
Out-of-plane CH bending(BP)		855/874	852/881
Ring breathing(BP)		1012	998
CC stretching(SQ)	1170	1138	
In-plane CH bending(BP)		1225	1217
CC stretching(between BP ring)		1290	1295
CN stretching(BP)		1518	1511
C = C stretching(in BP ring)		1609/1628	1595/1604/1619
C = O stretching(SQ)	1821		
CH stretching(BP)		3046/3065/3076/3089	2971/3006/3027/3052

*Due to the complete crystalline acoustic phonon at low wavenumber, we could not make appropriate assignments for the Raman bands lower than 300 cm^−1^.

### 3.2 Analysis of vibrational spectra of SQBP under compression

According to the previous experimental study of proton transfer in SQBP^47^, the color of SQBP changed from yellow to red when heated to 453K, and the differential scanning calorimeter (DSC) measurements revealed that the color change is accompanied by an energy absorption of 19.8 J/g. The same color changes can be induced by pressure^46, 58^. Both experimental and theoretical approaches, suggest that the color change is the result of double proton transfer in SQBP. In our investigation, above the pressure 1.5 GPa, the rectangular yellow crystal of SQBP turned into dark red, the optical microscopy images of the SQBP co-crystal in the DAC at various pressure can be seen at Figure S1 (Supporting Information). The UV-visible absorption spectra of SQBP are shown in Figure S2 (Supporting Information). A single absorption band is observed at 435 nm under ambient condition. This band shifts to 461 nm along with the formation of a new band at 551 nm as pressure increases to 1.5 GPa, which is consistent with the observed color change.

**Figure 3:**
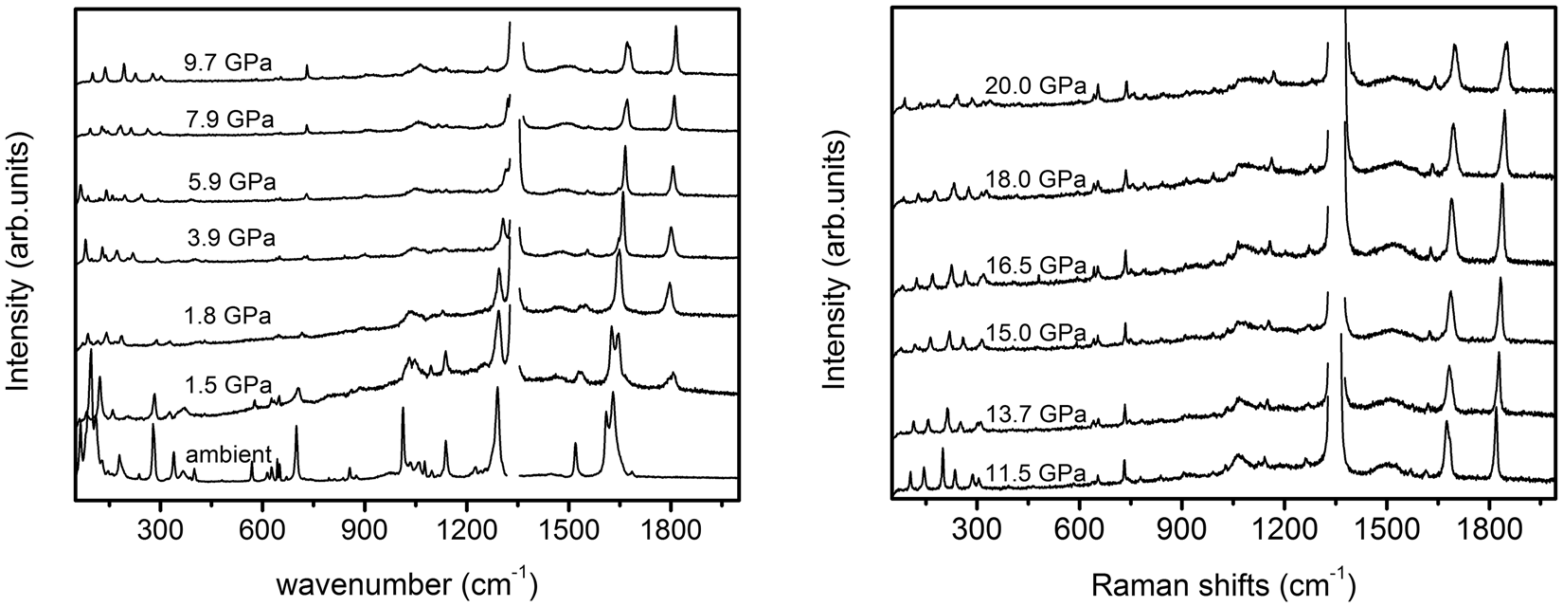
Selected high pressure Raman spectra of SQBP.

**Figure 4:**
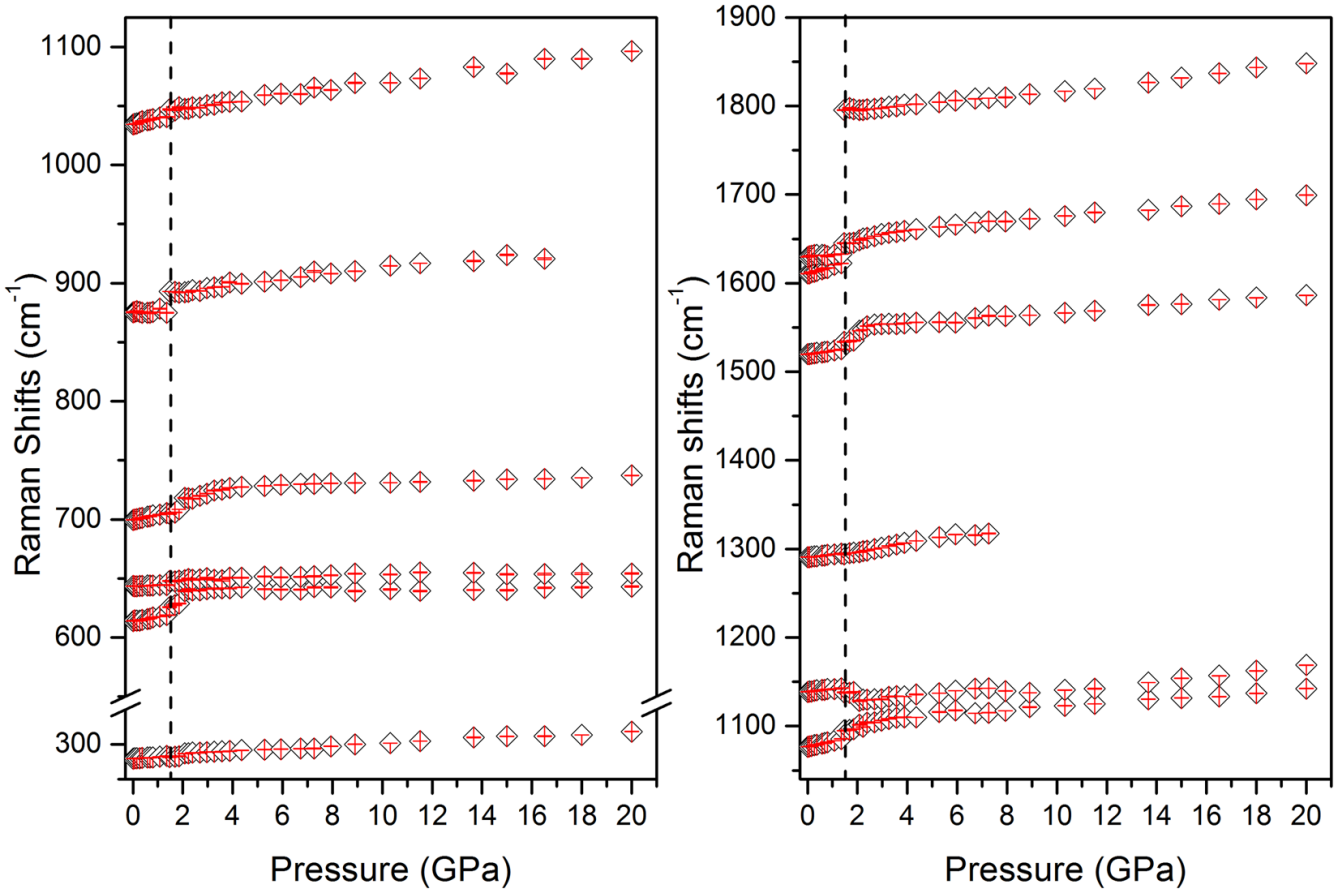
Frequency-pressure relationship of SQBP.

It has been reported that a double proton transfer from SQ^1+^BP^1−^ to SQ^2+^BP^2−^ can occur under external compression^46^. They casted a doubt about the conclusion of the same structure of crystal in high temperature and high pressure phase. However, due to the cracked crystal during the compression process, all attempts to index the crystal structure in high pressure phase failed. In order to solve this discrepancy, Raman spectroscopy was used to detect the structural changes during the compression process. *In-situ* high pressure Raman spectra of SQBP were measured up to 20 GPa, Selected Raman spectra of SQBP are shown in Fig. 3; Also, Raman measurements were conducted on decompression all the way down to the ambient pressure. The spectra are more or less similar to those of compression. The reversible Raman spectra indicate that SQBP is chemically stable up to 20 GPa. It suggests that there is no kinetic barrier during the decompression process similar with during the heat-cool down cycle^47^. As changes in response of the Raman mode with pressure is a good indicator of a possible phase transition^38–41, 53^, the Raman frequency shift as a function of pressure for SQBP is shown in Fig. 4, it can be found that the frequencies vary almost linearly with pressure. A monotonous red-shift of the vibrational frequency in Raman spectra is observed up to 20 GPa. The spectroscopic results indicate substantial changes of molecular configuration at 1.5 GPa from the abrupt change of splitting, disappearance, and appearance of some modes. It is concluded that the transition was the first order. On further compression, no obvious spectral evidence of first order phase transitions can be observed up to the applied highest pressure. Two different crystallographic phases are labeled in this pressure range, the solid phase below 1.5 GPa for phase I, and above 1.5 GPa for phase II. These results are further supported by the high pressure infrared absorption spectroscopy, the spectra and frequency-pressure curves are given in Figures S3 and S4 respectively (Supporting Information).

To understand the local structural changes of SQBP at high pressure, we performed *ab initio* calculations of SQBP up to 2.5GPa. Based on the calculated value, we plot the bond length of N-H and O-H changes as a function of pressure in Figure S5 (Supporting Information), It can be seen that the proton transfer from SQ to BP occurs at about 1.5 GPa suggests that the double proton transfer structure possess the lowest enthalpy under compression, and the first-order phase transition can be associate with the proton transfer^59^. For BP molecule, the two pyridine rings are not coplanar and form a dihedral angle of 34◦ at ambient condition, the calculated dihedral angle decreases with increasing pressure (shown in Figure S6 (Supporting Information)), which is similar to the structural change of BP at high temperature^46^. According to their analysis, the Uv-Visible absorption band associate with an intra-molecular electron transition from SQ π orbital to BP π* orbital. The decrease in dihedral angle between the pyridine rings enhances the π electron delocalization along the BP backbones.

After determining the co-crystal structure of SQBP at different pressure in the *ab initio* calculations, we plot the HOMO and LUMO orbitals of SQBP at ambient and 1.5Gpa respectively, which is shown in Figure S7 (Supporting Information). From the molecular orbital diagram, it can be seen that more nodes are present in the LUMO orbitals. And significant differences can be found from the Homo orbitals of SQBP co-crystal at ambient and 1.5 GPa. At ambient condition, the electrons in the HOMO orbital are located in the one ring of BP molecule, while in contrast, when the pressure increased to 1.5 GPa, the second proton in SQ transferred to BP molecule, which makes the electrons delocalization in the HOMO orbital of BP. As a result, the electrons can be delocalized through the extended backbones of proton donor and acceptor’s co-crystal. Thus the changes in electron density distribution should be responsible for the high pressure Uv-Visible absorption spectra.

### The observation of intensity enhancement of C = O stretching mode

As shown in Fig. 3, an intriguing phenomenon is the pressure-induced intensity enhancement of C = O stretching band at phase II. The C = O stretching band of SQBP can’t be observed at ambient condition. When the applied pressure increases to 1.5 GPa, the C = O band with a small intensity appears at 1795 cm^−1^ in the Raman spectrum of SQBP. The further compression will induce the intensity of C = O stretching mode to enhanced gradually, and there are three possible ways to explain this spectroscopic phenomenon.

Firstly, according to the previous analysis, double proton transfer occurs in the phase transition from phase I to phase II at 1.5 GPa, which increases the number of C = O bonds in each SQ molecule from three to four. Then it is reasonable to assume that there is a linear correlation between the Raman intensity and the number of the C = O bonds in SQ. However, this possibility can be ruled out because the intensity enhancement factor is greater than four thirds.

Secondly, as shown in Figure S2 (Supporting Information), the laser excitation line(532 nm) falls into the region of the electronic absorption of red crystal in phase II, and hence the resonance Raman effects should be taken into consideration, which can greatly lead to intensity enhancement of the Raman scattering^60, 61^. It has been reported that SQBP at higher temperature(453 K) has the same color with that at the pressure of 1.5 GPa^46^. If the Raman intensity enhancement is attributed to the resonance Raman effects, a similar phenomenon should be observed in the high temperature Raman spectrum of SQBP. The Raman spectra of SQBP at the pressure of 1.5 GPa and the temperature of 453K are shown in Figure S8 (Supporting Information). Because the C = O stretching band cannot be observed in the high temperature Raman spectrum of SQBP, we cannot simply attribute this intensity enhancement to the resonance Raman effects.

Thirdly, the sudden appearance of the carbonyl stretching band in phase II may rise from the changes in the molecular symmetry. As a result of the pressure-induced double proton transfer and phase transition, the SQ molecule in phase II contains four C = O double bonds, and it belongs to a high symmetry point group. Then it is reasonable to assume that the observation of C = O stretching Raman band is attributed to activation of a silent mode due to a change in molecular symmetry.

**Figure 5:**
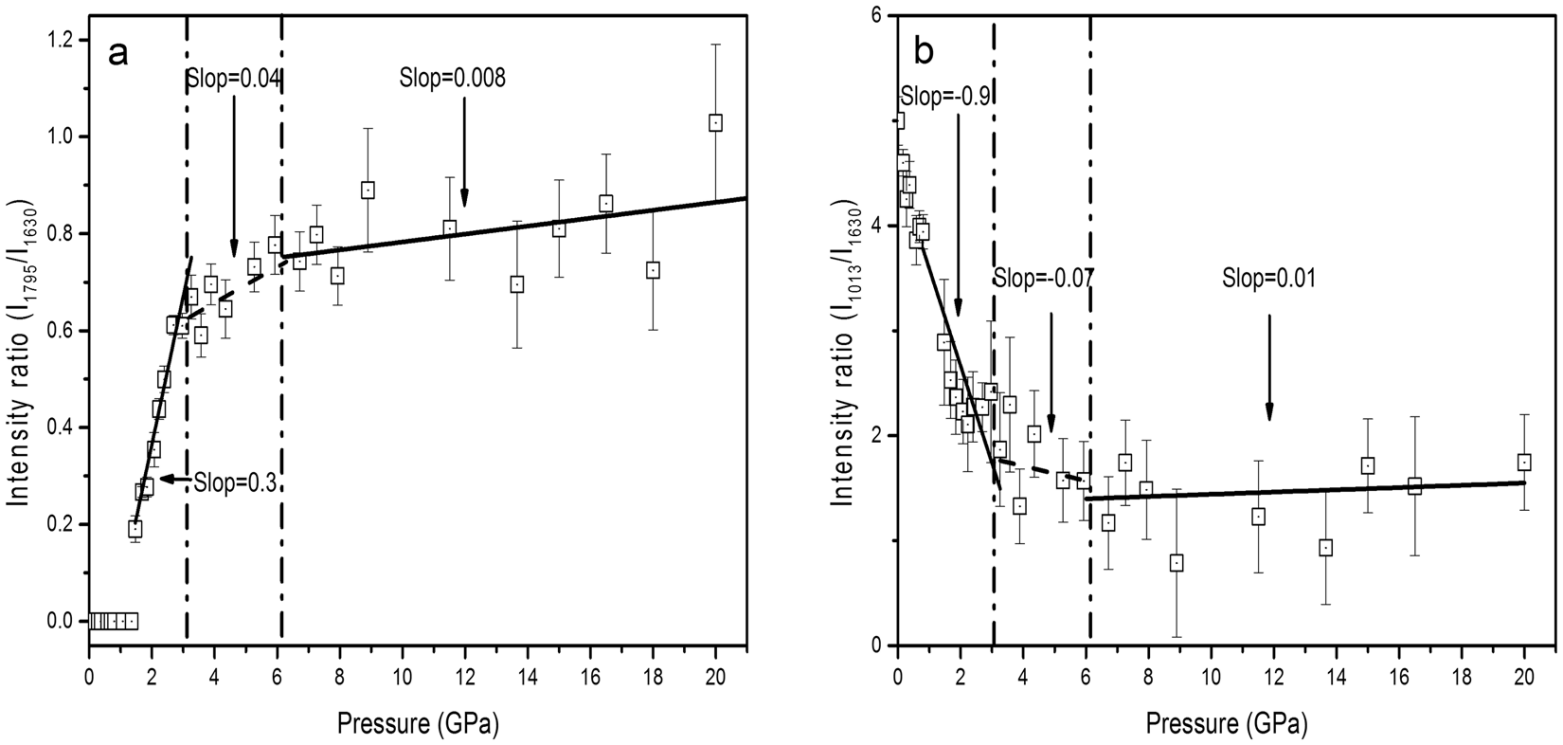
Intensity ratio -pressure curve of SQBP (**a**),1795 cm^−1^ to 1630cm^−1^ and (**b**), 1013 cm^−1^ to 1630 cm^−1^.

As shown in Fig. 3, the intensity of C = O stretching band is increased by further compression when the pressure is higher than 1.5 GPa. Theoretical and experimental approaches show that the relative change in the Raman intensity is associated with some special molecular structural changes, and this method is especially useful for detection of planar and non-planar aromatic molecules. Take oligo-*p*-phenylenes^50, 51, 62, 63^, as example, the major structural change in response to high compression tends to transform from non-planar toward planar shape, and the resulting delocalization of π electrons make the intensities of some Raman bands enhance remarkably. Furthermore, the sudden slope change in intensity-pressure curves can be associated with higher-order phase transition^61^ Fig. 5a displays an intensity ratio (1795 cm^−1^ to 1630cm^−1^)-pressure curve of SQBP. The 1630 cm^−1^ Raman band is attributed to the C-C stretching vibration mode of BP. The line was created by curve fitting using linear regression with the slope of 0.3s at pressure below 3 GPa. The further compression higher than 6 GPa induces a weak intensity enhancement of the C = O stretching Raman line to give a slope of 0.008. The proton acceptor-BP possesses a non-planar aromatic molecular geometry under ambient conditions. It has been reported that the intensity ratio between ring breathing and C-C stretching mode can correlate with the dihedral angle between two pyridine ring in BP^64–67^. The smaller dihedral angle is, the lower intensity ratio of ring breathing to C-C stretching mode is. Figure 5b shows the intensity ratio(1013 cm^−1^ to 1630 cm^−1^) -pressure curve, where the 1013 and 1630 cm^−1^ bands are assigned to ring breathing and C-C stretching mode of BP, respectively. The curve experiences a sudden slope change at about 3 GPa, which is consistent with the changing trends observed in Fig. 5a. Thus, as the intensity behaviors shown in Fig. 5a and b. it is believed that a higher-order phase transition may occur in the pressure range of 3~ 6 GPa, and the higher-order phase transition is related to the planarization of proton acceptor-BP. In the pressure range from 1.5 to 3 GPa, the decrease in dihedral angle between the pyridine rings enhance π electron delocalization along the BP backbones, which may further induce a significant increase in the intensity of the C = O stretching band of proton donor-SQ. At pressures higher than 6 GPa, the planar BP structure does not cause any further π electron delocalization, which does not results in the bigger intensity enhancement of C = O stretching mode.

In Fig. 3, when the pressure increased to 1.5 GPa, an additional broad band appears at about 1500 cm^−1^ in the Raman spectrum of SQBP, and further compression will induce the intensity of the broad band enhances gradually, the broad feature of the new arising band should be attributed to vibrational mode of square acids, It has the similar pressure-induced change tendency with C = O stretching mode. Therefore, this broad feature should be associated with the high symmetry structure of square acids at high pressure phase.

## Conclusions

Through comprehensive Raman spectroscopy studies, the detailed structural characteristic of proton acceptor-BP, proton donor-SQ and their corresponding co-crystal-SQBP are discussed. *In-situ* high pressure Raman spectra of SQBP have been measured up to 20 GPa, a first order phase transition associated with double proton transfer in the hydrogen bonds of co-crystal is concluded at about 1.5 GPa. Additionally, a higher-order phase transition with planar BP molecular structure is predicted in the pressure range of 3~6 GPa from the analysis of relative Raman intensity. The delocalization of π electron arising from the decrease in dihedral angle between two pyridine rings in BP makes the intensity enhancement of C = O stretching mode in SQ.

## Electronic supplementary material

Supplementary information
