# Supplementary material for: High Pressure Spectroscopic Investigation on Proton Transfer in Squaric Acid and 4,4′-Bipyridine Co-crystal
Source: Sci Rep. 2017 Jul 5;7:4677. doi: 10.1038/s41598-017-04980-3 (PMC5498627; doi:10.1038/s41598-017-04980-3)
Supplement: Supplementary file 1 — Supplementary information [file 41598_2017_4980_MOESM1_ESM.doc]

**High Pressure Spectroscopic Investigation on Proton Transfer in Squaric Acid and 4,4’-Bipyridine Co-crystal**

Zhiwei Ma, Juntao Li, Chunyu Liu, Chenglin Sun and Mi Zhou[[1]](#footnote-2)

*Key Laboratory of Physics and Technology for Advanced Batteries (Ministry of Education), College of Physics Jilin University, Changchun 130012, P.R. China*


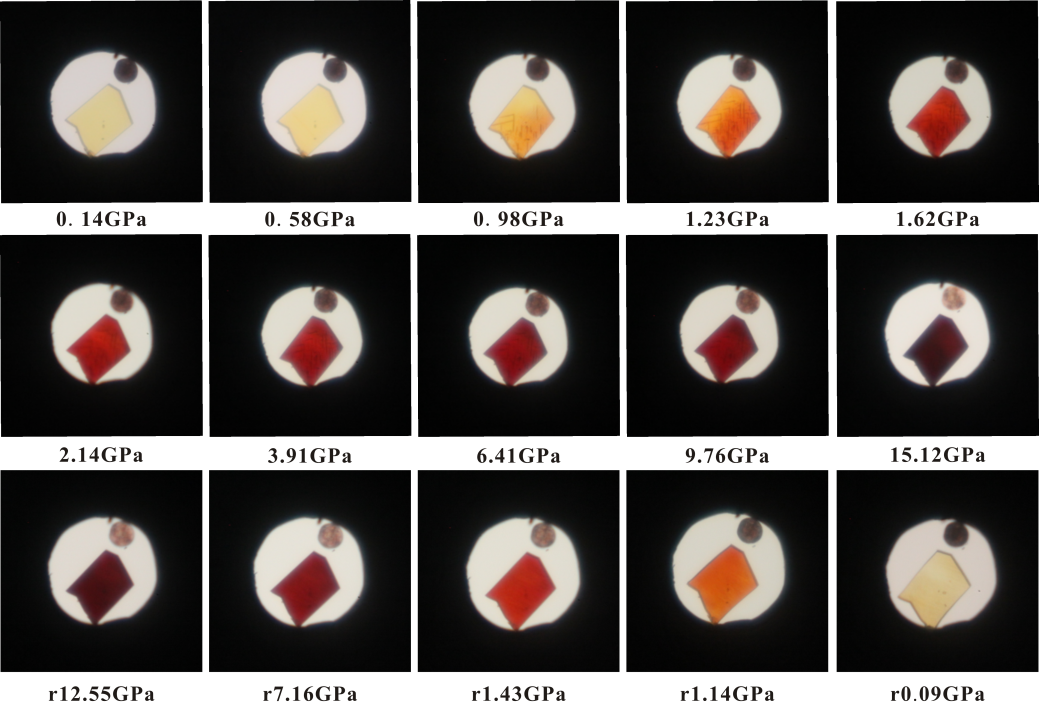


Figure S1. Optical microscopy images of the SQBP co-crystal at various pressure(r indicates the release process)


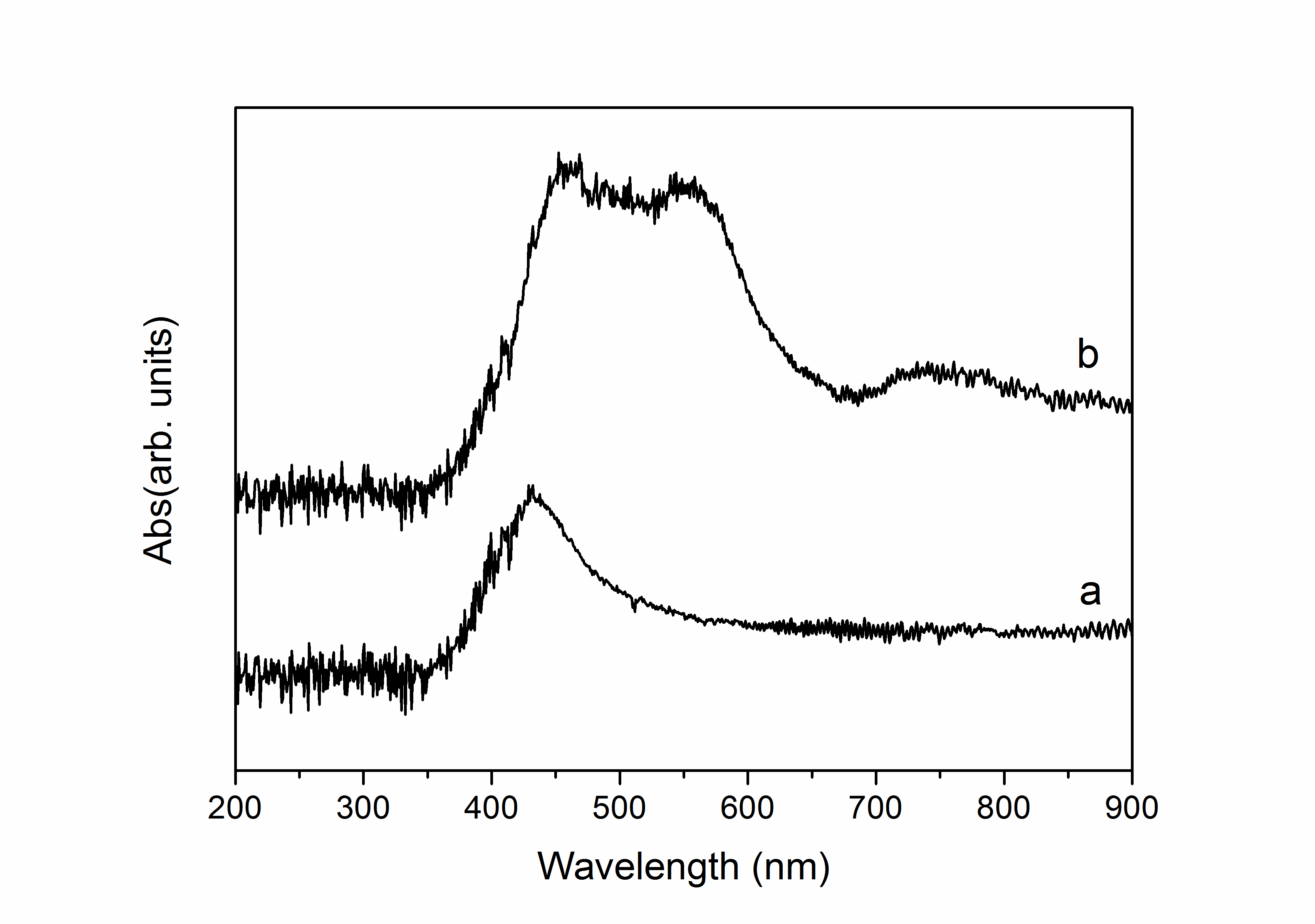


Figure. S2. Uv-Visible absorption spectra of SQBP at ambient(a) and 1.5 GPa(b)


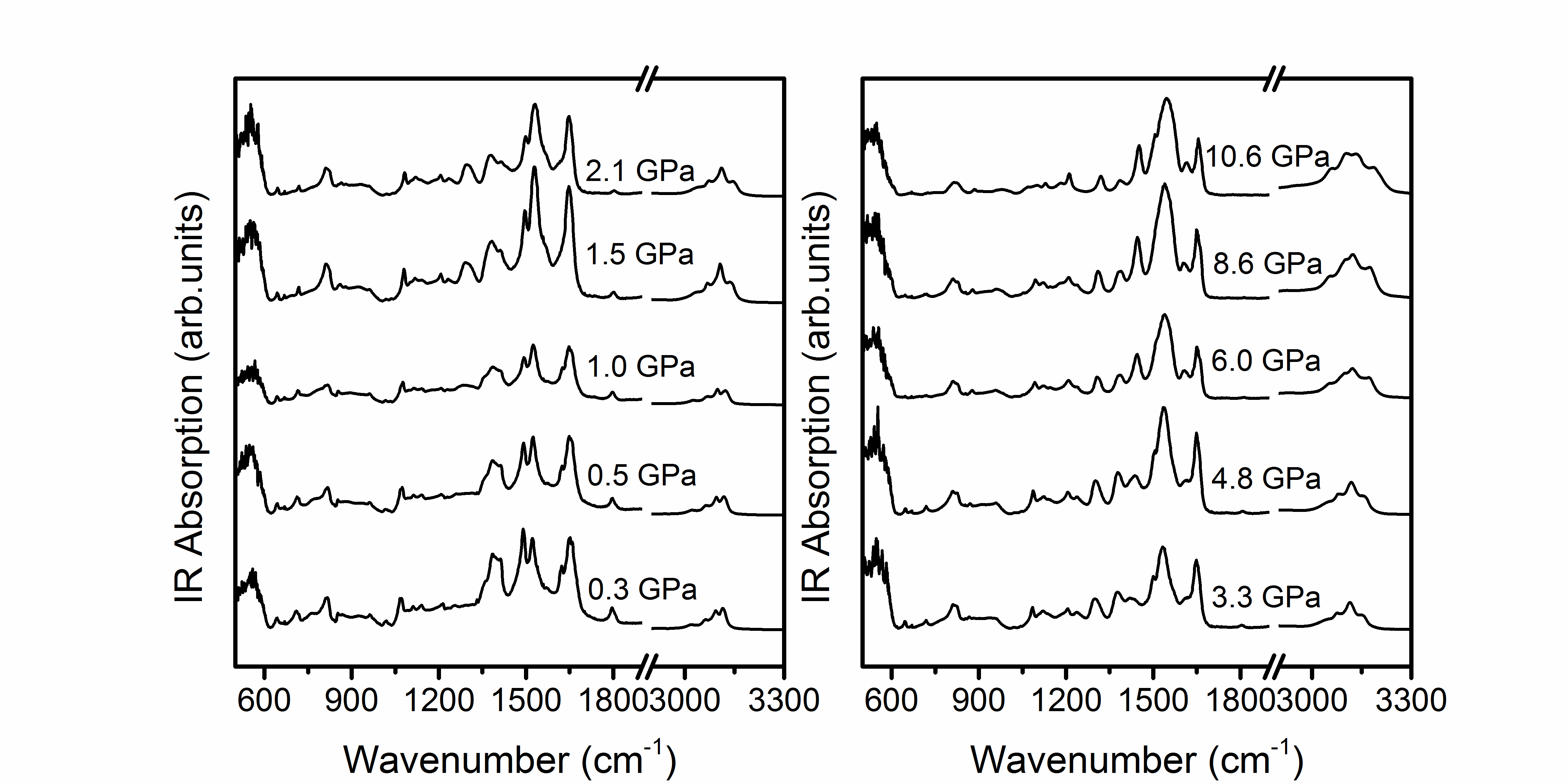


Figure S3. High pressure infrared absorption spectra of SQBP


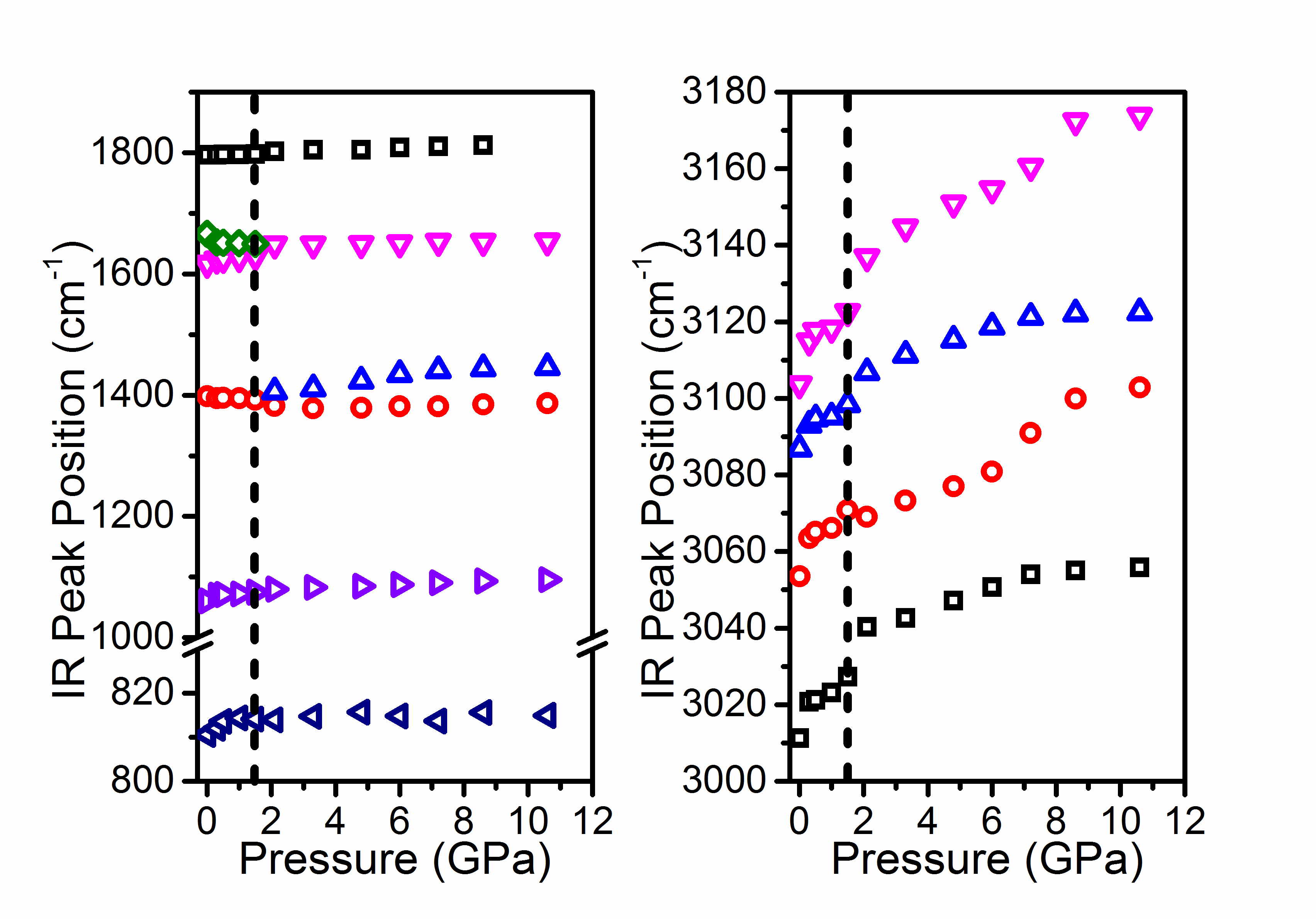


Figure S4.Frequency-pressure relationships of IR spectra of SQBP


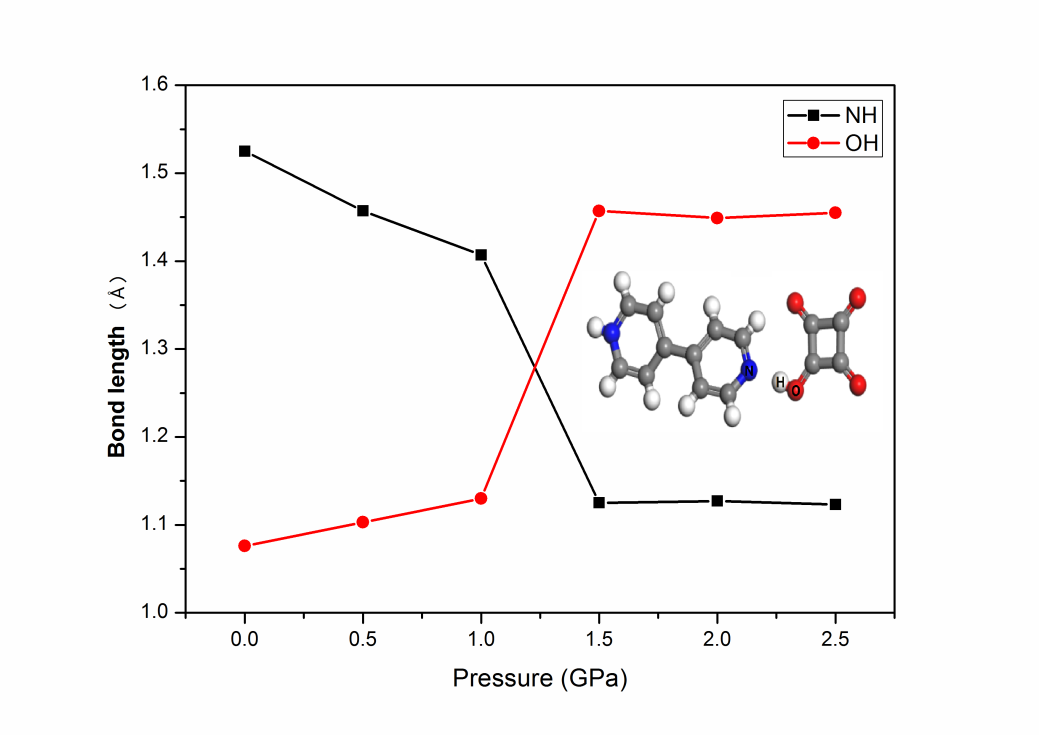


Figure S5. The bond length of N-H and O-H under pressure


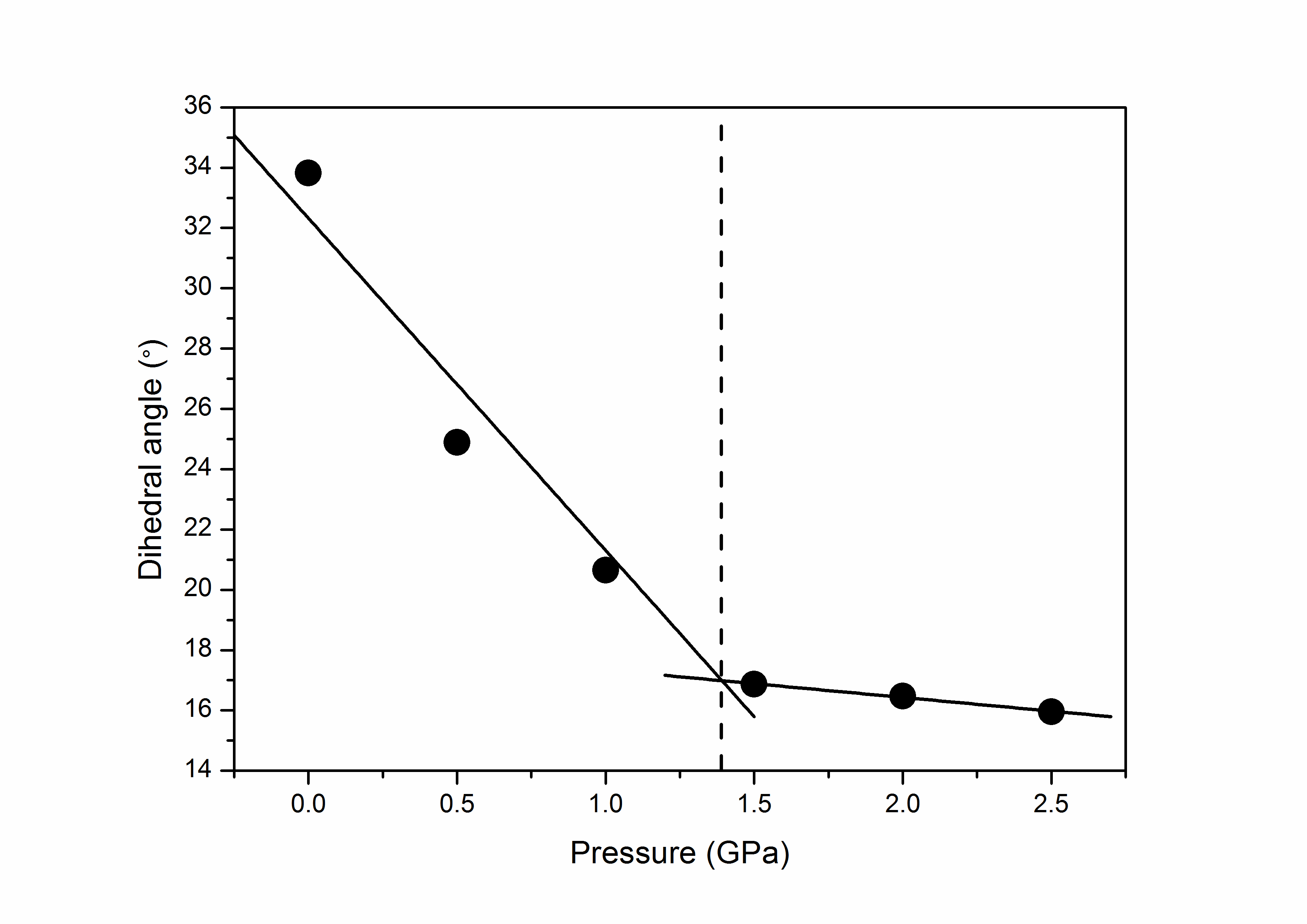


Figure S6. Dihedral angle between two pyridine rings in BP at high pressure


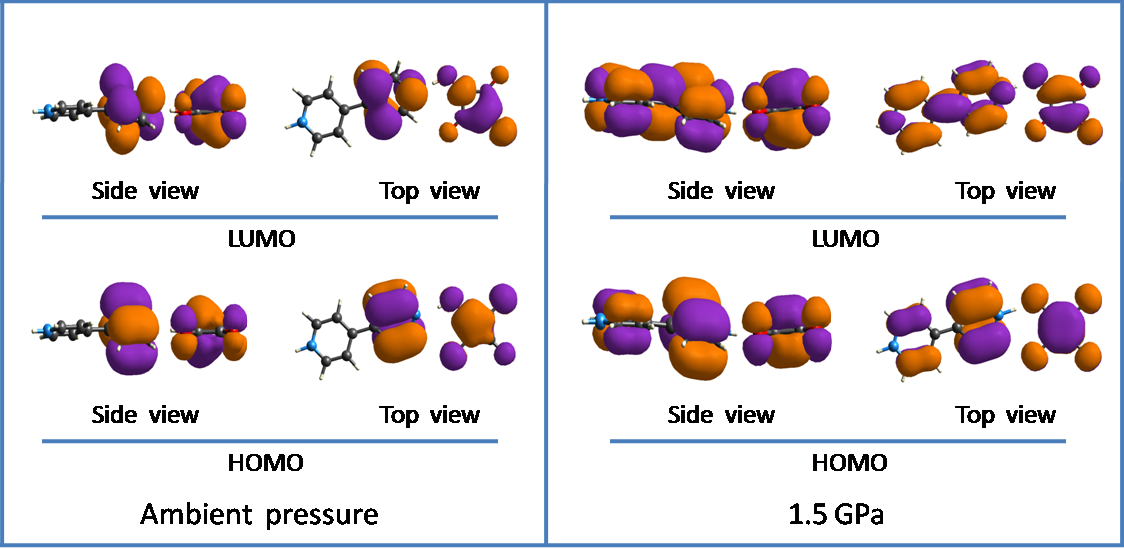


Figure S7. HOMO and LUMO of SQBP at ambient and 1.5 GPa


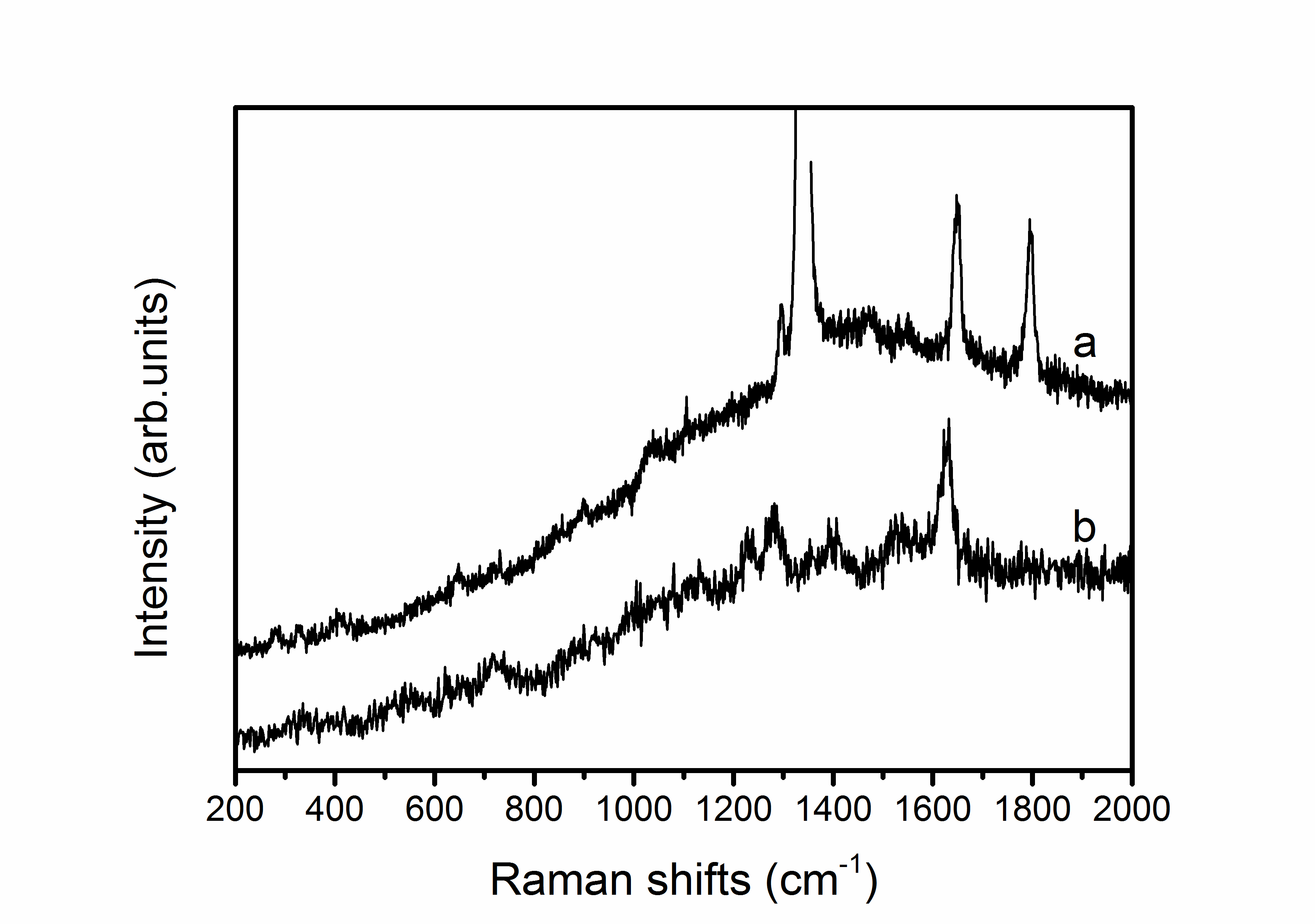


Figure S8. The Raman spectra of SQBP at the pressure of 1.5 GPa (a) and the temperature of 453K(b)

1. **Aurthor to whom correspondence should be addressed. Electronic mail:mzhou@jlu.edu.cn** [↑](#footnote-ref-2)
